# Exploring cadmium-binding proteins in Japanese scallops* Mizuhopecten yessoensis*: isolation and characterization

**DOI:** 10.1007/s11356-025-36799-1

**Published:** 2025-08-05

**Authors:** Zehua Zheng, Yuto Namikawa, Yugo Kato, Peng Lu, Lumi Negishi, Hitoshi Kurumizaka, Koji Nagata, Michio Suzuki

**Affiliations:** 1 Department of Applied Biological Chemistry, Graduate School of Agricultural and Life Sciences, The University of Tokyo, 1-1-1 Yayoi, Bunkyo-Ku, Tokyo, 113-8657 Japan; 2 Bone Marrow Transplantation Center of the First Affiliated Hospital & Liangzhu Laboratory, Zhejiang University School of Medicine, Hangzhou, China; 3 Future Food Laboratory, Innovation Center of Yangtze River Delta, Zhejiang University, Jiashan County, No. 828, Zhongxing Road, Xitang Town, Jiaxing City, Zhejiang Province China; 4 Institute for Quantitative Biosciences, The University of Tokyo, 1-1-1 Yayoi, Bunkyo-Ku, Tokyo, 113-0032 Japan

**Keywords:** *Mizuhopecten yessoensis*, Midgut gland, Cd-binding substance, Cadmium, Identification, Purification, Scallop, Accumulation

## Abstract

Scallops (*Mizuhopecten yessoensis*) accumulate abnormal levels of cadmium (Cd) in their bodies; however, the chemical form of Cd in vivo remains unclear. In this study, Cd concentrations in various scallop tissues were measured, revealing that Cd predominantly accumulated as a water-soluble component within the midgut gland. High-performance liquid chromatography combined with the post-column tetraphenylporphyrin tetrasulfonic acid (TPPS) experiment revealed that the molecular weight of the Cd-binding substances was approximately 44 kDa. Moreover, Cd-immobilized affinity chromatography and CdS precipitation identified *M. yessoensis* meprin A subunit alpha-like (myMEP1A) as a Cd-binding protein. The myMEP1A gene was specifically expressed in the midgut gland. TPPS measurements indicated that myMEP1A exhibited strong Cd-binding activity. These results suggested that myMEP1A is a protein responsible for Cd accumulation in the midgut gland.

**Supplementary Information:**

The online version contains supplementary material available at 10.1007/s11356-025-36799-1.

## Introduction

Scallops, belonging to the phylum Mollusca, inhabit shallow waters with sandy bottoms. They primarily feed on algae (Shumway and Parsons 2006). Scallops are globally distributed in habitats such as the Kamchatka Peninsula, Kuril Islands, Sakhalin, and Primorskaya Oblast in Russia; Hokkaido and Tohoku regions in Japan; and the northern part of the Korean Peninsula. They have also been cultivated in certain regions of China and the United States. *Mizuhopecten yessoensis*, a scallop that is abundant in Japan, is a prominent target for harvesting because of its high consumption demand. According to the survey on fishery production of aquatic animals in marine areas, categorized by the ISSCAAP division and primary species items, *M. yessoensis* showed the second largest catch volume among molluscs. The reported harvest in Japan reached 497,000 tons in 2020, a remarkable 50% increase from the previous year (FAO 2023). Notably, the highest production in Japan occurred in Hokkaido (Ministry of Agriculture, Forestry and Fisheries, Japan 2023). These scallops are not only important to fisheries but are also distinguished by their nutritional composition. They have high protein content, constituting 55.6% of their maximum dry weight. Additionally, they are rich in polyunsaturated fatty acids, comprising approximately 9–45% of their total fatty acid content. Furthermore, *M. yessoensis* has a versatile role in culinary applications and are a key ingredient in many recipes. Scallops are widely used as flavor enhancers and seasoning agents, contributing to their diverse culinary uses.

The artificial release of cadmium (Cd) into the environment has been ongoing for decades owing to the impurities in metal refining and the burning of fossil fuels (Patra et al. 2011). Cd is highly toxic to humans and has been associated with the development of itai-itai disease, a severe symptom resulting from the consumption of crops contaminated with Cd. Itai-itai disease is widely recognized as one of the four major pollution-related illnesses in Japan. Consequently, international interest in the regulation of Cd contamination in food products is significant. According to the Codex Alimentarius, the acceptable Cd concentration in various foods includes limits of approximately 0.4 ppm for rice, 2 ppm for shellfish, and 0.8 ppm for chocolate.

Cd belongs to group 12 of the periodic table and shares similarities in chemical properties with zinc (Zn), an essential trace element. Cd^2+^ can be replaced with Zn^2+^ in biological systems and bind to thiol groups, thereby inhibiting enzymatic activity. Cd substitution for Zn is a source of Cd toxicity, affecting the proteins involved in the DNA base mismatch repair mechanism (McMurray and Tainer 2003). Cd^2+^ is classified as a soft metal according to the Hard and Soft Acids and Bases (HSAB) theory, allowing it to replace other soft metals such as Cu^+^ and Fe^2+^. This can lead to electron transfer, the generation of oxygen radicals through the redox of free metal ions, and the production of highly reactive radical species, resulting in detrimental effects such as lipid peroxidation and DNA sequence damage in living organisms (Muñoz et al. 2008; Stohs 1995). Additionally, Cd^2+^ shares a similar ionic radius and charge density with Ca^2+^ and can inhibit Ca^2+^-ATPase, which regulates Ca^2+^ efflux (Choong et al. 2014).

Bivalves, including scallops, can accumulate metals, such as Cu, Cd, and Zn, often tolerating high concentrations of metals without displaying any obvious signs of toxicity (Pringle et al. 1968). Almost all bivalves exhibit high uptake rates of metals when compared to their excretion rates, resulting in the accumulation of heavy metals in their bodies, even when metals are not present at high concentrations in the surrounding water (Lu et al. 2017). Cd is primarily taken up through filter-feeding, via both passive diffusion and active transport (Xu and Yu 2024). After absorption, Cd is transported via the hemolymph and accumulates mainly in the midgut gland. Inside cells, Cd binds to thiol-rich molecules such as glutathione, cysteine, and Cd-binding proteins, facilitating Cd detoxification. While some Cd may be excreted from the body, Cd can accumulate in scallop tissues over time. Cd exposure can lead to oxidative stress, immune suppression, and metabolic disruption in molluscs, where these toxic effects are often dose- and tissue-dependent (Bao et al. 2022; Li et al. 2023; Liu et al. 2023; Wu et al. 2023). In particular, exposure to multiple heavy metals induces reproductive toxicity in bivalves (Marinaro et al. 2024). Thus, bivalves possess heavy metal detoxification systems in specific tissues. Cd accumulation in bivalves is affected by seasonal changes (Bel’cheva et al. 2002; Piscopo 2019). However, the mechanisms underlying the accumulation of heavy metals in scallops remain unclear. Furthermore, scallops accumulate and retain higher levels of Cd than oysters and mussels (Bendell 2009; Cranford 2006).

Because scallops can withstand high concentrations of heavy metals, they are hypothesized to possess biological mechanisms for detoxifying these metals. Metallothionein is a well-known low-molecular weight protein with a cysteine-rich structure, capable of binding to and detoxifying heavy metals (Hamer 1986). Cd^2+^ tetrahedrally coordinates to cysteine thiolate ligands of metallothionein (Boulanger et al. 1983). Although metallothionein-like low-molecular and high molecular weight proteins have been reported in the Cd-containing fraction, distinguishing which proteins are related to Cd accumulation and determining the whole amino acid sequences has been challenging (Evtushenko et al. 1986; Gao et al. 2016). Thus, the purpose of this study was to isolate, identify, and characterize potential Cd-binding substances in *M. yessoensis* with the aim of gaining a better understanding of the mechanisms underlying Cd accumulation in scallops.

## Materials and methods

### Scallop anatomy

Four-year-old *Mizuhopecten yessoensis* were collected in July, during the summer season, from aquaculture sites near Rebun Island, Hokkaido, Japan. They were bred in artificial seawater for one day to reduce stress caused by environmental changes. The gonad, midgut gland, mantle, gill, and adductor muscle were dissected from the living body. Each tissue was frozen in liquid nitrogen immediately after excision, weighed, and subsequently stored at –80 °C.

### Separation and determination of Cd-containing substances from scallops

Each tissue was dried in an 80 °C dryer, and 100 mg of dried tissues were mixed with 1 mL of concentrated nitric acid (for analysis of poisonous metals, FUJIFILM Wako) in a Teflon-based container. Wet ash decomposition was performed at 150 °C for 10 h. The decomposed product was diluted up to 10 mL with 0.1 M nitric acid and used as a measurement sample. ICP-OES (SPS3500, SII Nano Technologies, Hitachi) was used for the measurement. The metal concentration of Cd (wavelength: 214.438 nm) was measured in tissues from three different individuals. Statistical differences between the metal concentrations were assessed using the Tukey–Kramer multiple comparison method at the *p* < 0.05 level.

The midgut gland of the scallop was frozen and crushed using liquid nitrogen. Extraction buffer (20 mM HEPES–NaOH, pH 7.4) with a weight ratio of five times was added to the pulverized product, mixed well with a vortex mixer, and extracted overnight at 4 °C. The extract was centrifuged at 4 °C and 10,000 g for 10 min, and the supernatant was ultra-centrifuged (himac CP56, Koki Holdings Co., Ltd.) at 4 °C and 100,000 g for 2 h. The sample obtained after centrifugation was divided into three layers: an oil layer, supernatant, and precipitate. Wet ash decomposition was performed for the three layers, followed by the measurement of Cd concentration using inductively coupled plasma optical emission spectrometer (ICP-OES).

### Detection of Cd-binding protein

#### HPLC combined with post-column TPPS method

The post-column detection of Cd-binding substances employed a modified tetraphenylporphyrin tetrasulfonic acid (TPPS) method (Chen et al. 2020). The method aimed to detect and quantify Cd-binding substances in the midgut gland extract. The midgut gland extract was ultrafiltrated and filtered through a 0.22 μm filter to obtain a purified sample. Then, high-performance liquid chromatography (HPLC) was conducted using a YMC Pack Diol-120 gel filtration column (4.6 mm × 300 mm). The mobile phase consisted of 20 mM HEPES–NaOH (pH 7.0) and 0.2 M sodium sulfate. Organic molecules were detected by measuring the absorbance at a wavelength of 225 nm. The flow rate was maintained at 1.0 mL/min, and the column temperature was set to 40 °C. For the post-column TPPS detection, a solution containing 2 μM TPPS, 6 μM cadmium sulfate, 50 mM HEPES–NaOH (pH 8.0), and 0.1% sodium dodecyl sulfate (SDS) was used. Cd-binding substances were detected at a wavelength of 414 nm.

#### Cd-immobilized affinity chromatography method

Cd-immobilized metal affinity chromatography was performed to separate the Cd-binding substance. The addition of excess Cd to Cd-binding substances induced the formation of Cd-complexes that do not interact with the Cd-immobilized affinity resin. Then, Cd-binding substances were distinguished depending on the presence or absence of the addition of excess Cd to the midgut gland extract. The midgut gland extract was mixed with 1 mM cadmium sulfate and passed through a 0.45 µm filter to remove precipitates. Subsequently, 10 mL of the affinity resin was equilibrated with 5 mL of 0.2 M cadmium sulfate. The unbound Cd was washed out with 100 mL of ultrapure water, 30 mL of elution buffer (20 mM sodium phosphate (pH 3.0), 0.2 M sodium sulfate), and 30 mL of binding buffer (20 mM sodium phosphate (pH 7.0), 0.2 M sodium sulfate). Next, 10 mL of the midgut gland extract with and without 1 mM cadmium sulfate was loaded onto the equilibrated resin. To prevent non-specific interactions, the column was washed with 50 mL of binding buffer. Finally, the Cd-binding substance was eluted with 50 mL of elution buffer. Eluate was concentrated and desalted with ultrapure water via ultrafiltration (Amicon Ultra 10 kDa MWCO, Merck). The final protein concentrations were measured using the BCA method.

#### CdS precipitation method

The midgut gland extract containing 1 mM of cadmium sulfate was prepared. Sodium sulfide was added to the extract to achieve final concentrations of 0 and 1 mM. The solutions were centrifuged at 4 °C and 10,000 g for 10 min. The supernatant was passed through a 0.45 µm filter.

#### SDS-PAGE analysis

Sodium dodecyl sulfate–polyacrylamide gel electrophoresis (SDS-PAGE) analysis was performed using 10% gels to analyze the proteins obtained via the HPLC combined with post-column TPPS experiment, Cd-immobilized affinity chromatography, and CdS precipitation. After electrophoresis, the gel was stained using silver staining method according to the manufacturer’s protocol.

### Identification of Cd-binding proteins using LC–MS/MS

The stained gel was washed with ultrapure water, and the bands obtained using Cd-immobilized affinity chromatography and CdS precipitation were excised. Proteins from the excised gel were digested by trypsin following a previously described method (Zheng et al. 2021). The tryptic digest underwent liquid chromatography-tandem mass spectrometry (LC–MS/MS), and the results were analyzed via peptide mass fingerprinting using Proteome Discoverer 2.1 (Thermo Fisher Scientific) combined with the genome database specific to *M. yessoensis* (Schoch et al. 2020).

### Tissue-specific expression analysis of myMEP1A

#### Scallop total RNA extraction and cDNA synthesis

The gonad, midgut gland, mantle, gills, and adductor muscle were dissected from the living body of scallops, frozen in liquid nitrogen, and homogenized using a mortar. Next, 50 mg of tissues were transferred to a 1.5 mL tube containing 1 mL of RNA extraction reagent Sepasol® RNA I Super G (nacalai tesque). After vortexing, the mixtures were incubated at 4 °C for 5 min. After 200 µL of chloroform was added, the mixture was incubated at room temperature for 3 min and centrifuged at 4 °C and 12,000 g for 15 min. The supernatant was mixed with 500 µL of 2-propanol, incubated at room temperature for 10 min, and centrifuged at 4 °C and 12,000 g for 15 min. The precipitates were homogenized in 1 mL of 75% ethanol, and the mixture was centrifuged at 4 °C and 12,000 g for 5 min. The precipitates were recovered with TE buffer to obtain the total RNA. The first-strand cDNA was synthesized from the extracted total RNA using the PrimeScript™ RT reagent kit (Perfect Real Time) (Takara Bio) according to the manufacturer’s protocol.

#### Quantification of expression levels of myMEP1A

*Mizuhopecten yessoensis* meprin A subunit alpha-like (myMEP1A)-F, myMEP1A-R, actin-F, and actin R primers were designed with Primer BLAST to amplify 500 bp of DNA (Table S1) using polymerase chain reaction (PCR). Actin was used as a housekeeping gene. TaKaRa Ex Taq (Takara Bio) was used for PCR according to the manufacturer’s instructions. The synthesized first-strand cDNA was used as a template for PCR. The PCR program was as follows: 35 cycles of 30 s at 94 °C (5 min for the first cycle), 30 s at 55 °C, and 2 min at 72 °C (7 min for the last cycle). The PCR products were subjected to agarose gel electrophoresis, and the band intensity was analyzed using ImageJ software. Experiments were conducted in triplicate to ensure reproducibility. Statistical differences of relative expression levels were assessed using the Tukey–Kramer multiple comparison method at the *p* < 0.05 level.

### Preparation of recombinant myMEP1A (r-myMEP1A)

#### Preparation of recombinant vector by In-Fusion method

The linearized pET-44a (+) gene was prepared via PCR from a pET-44a (+) empty vector using the designed primer (Table S2). Dpn I treatment was performed overnight at 37 °C to remove the template pET-44a (+) empty vector from the PCR product. The Dpn I-treated solution was mixed with one-tenth volume of 3 M sodium acetate and 2.5 volumes of ethanol, vortexed, and incubated at –20 °C for 1 h. The supernatant was removed after centrifugation at 15,000 g at 4 °C for 30 min. The precipitate was diluted with 70% ethanol and centrifuged at 15,000 g at 4 °C for 2 min. The supernatant was completely removed by air-drying for 5 min. The precipitate was dissolved in TE buffer (10 mM Tris–HCl (pH 8.0) and 1 mM ethylenediaminetetraacetic acid) to prepare a linearized pET-44a (+) solution.

The myMEP1A gene was amplified from the cDNA of the midgut gland via PCR using the designed primer (Table S2). Next, 5 µL of the PCR product was mixed with 2 µL of Cloning Enhancer (Takara Bio). Then, the mixture was incubated at 37 °C for 15 min and 80 °C for 15 min. The In-Fusion reaction solution was prepared using the linearized pET-44a (+) and the myMEP1A gene. The solution was incubated at 50 °C for 15 min and placed on ice for In-Fusion cloning.

#### Overexpression of r-myMEP1A

Then, 1 µL of 100 ng/µL In-Fusion product was added to 100 µL of *Escherichia coli* BL21 (DE3) competent cells. The cells were incubated on ice for 30 min and heat-shocked at 42 °C for 40 s. After 300 µL of SOC medium was added, the cells were incubated at 37 °C for 1 h. The cells were plated on a LB plate containing ampicillin and incubated overnight at 37 °C.

The bacterial colony on the LB plate was transferred to 3 mL of LB medium containing 50 µg/mL ampicillin. The medium was pre-cultured overnight, transferred to 1 L of LB medium, and cultured until OD_600_ reached approximately 0.5. Protein overexpression was induced by adding isopropyl β-D-1-thiogalactopyranoside (IPTG) to the medium to final concentrations of 10, 100, and 1,000 µM. IPTG induction was performed at 37 °C for 2 h, 16 °C for 7 h, and 5 °C for 24 h. Then, cells were collected by centrifugation, sonicated, and separated into soluble fraction and inclusion body. Proteins in the inclusion body were solubilized using 8 M urea. The soluble fraction and insoluble fraction were subjected to SDS-PAGE to confirm the optimal condition of IPTG induction.

#### Purification of r-myMEP1A

The soluble fraction was applied to a Ni-affinity column equilibrated with 20 mM Tris–HCl (pH 8.0)/20 mM imidazole/0.5 M NaCl. The flow-through fraction was collected. Then, proteins were eluted with increased concentrations of imidazole at 20, 50, 100, 300, and 500 mM. The eluate was concentrated and desalted with 20 mM Tris–HCl (pH 8.0) via ultrafiltration (Amicon Ultra 30 kDa MWCO, Merck) followed by His-tag cleavage with thrombin (Cytiva) according to the manufacturer’s protocol. The purification and His-tag cleavage were confirmed by SDS-PAGE.

### Binding experiment of Cd and r-myMEP1A using TPPS

The solution containing 4 µg/mL protein, 50 mM HEPES–NaOH (pH 8.0), 6 μM cadmium sulfate, 2 μM TPPS, and 0.1% SDS was prepared. The proteins used in this experiment were r-myMEP1A, r-myMEP1A with His-tag, and bovine serum albumin (BSA). Ultrapure water was used as a negative control. The UV/vis spectra of the mixture were measured at a 400–450 nm wavelength.

### Cd-binding site prediction and sequential alignment of myMEP1A

The tertiary structure of myMEP1A without a signal peptide sequence was modelled using SWISS-MODEL server (Bienert et al. 2017; Waterhouse et al. 2018). Human meprin alpha (PDB ID: 7UAE), which contains a zinc ion and two calcium ions, was used as a template for modeling (Bayly-Jones et al. 2022). The Cd-binding site was predicted by referring to the metal binding mode of 7UAE. The protein structure was visualized using UCSF chimera (Pettersen et al. 2004).

The amino acid sequence of myMEP1A was compared to the MEP1A of other molluscs using CLUSTALW (Larkin et al. 2007). The aligned amino acid sequences were MEP1A (meprin A subunit alpha-like) of *Argopecten irradians* [XP_069106106.1], *Magallana gigas* [XP_034302681.1], *Mytilus edulis* [XP_071142742.1], *Ostrea edulis* [XP_048729354.2], *Pecten maximus* [XP_033727200.1], and *Ylistrum balloti* [XP_060082176.1]. The alignment image was visualized using ESPript 3.0 (Robert and Gouet 2014).

## Results

### Cd concentration in each tissue

**Fig. 1:**
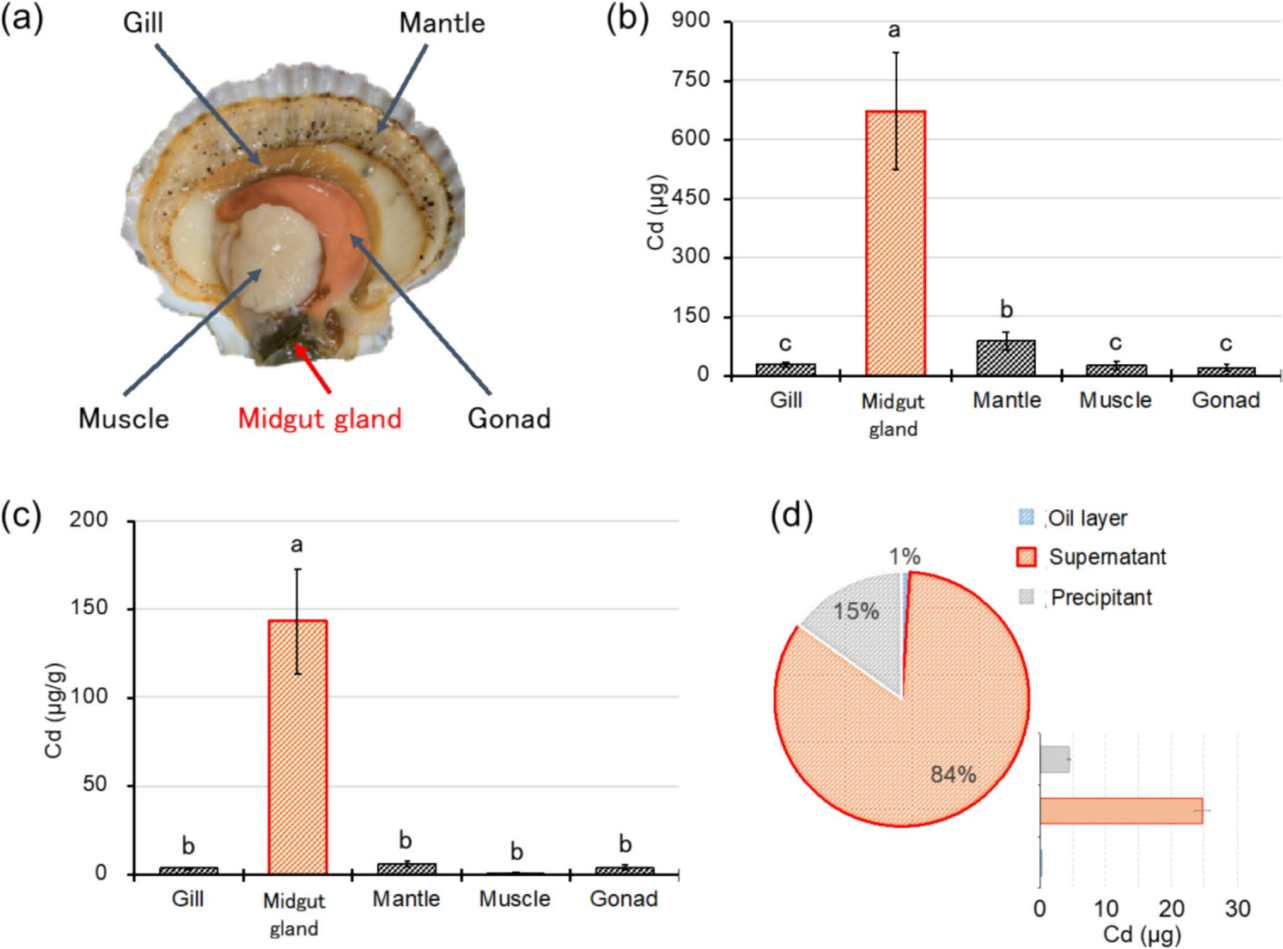
Distribution of Cd in each layer of the midgut gland extract. (**a**) Five tissues: the gill, midgut gland, mantle, adductor muscle, and gonad, were dissected from the scallops. (**b**) Total amount of Cd in each scallop tissues. Error bars represent the standard deviation (n = 3); mean values with different lower-case letters (e.g., a–c) are statistically different from one another (Tukey–Kramer multiple comparison test, *p* < 0.05). (**c**) The Cd concentration per tissue weights. Error bars represent the standard deviation (n = 3); mean values with different lower-case letters (e.g., a–c) are statistically different from one another (Tukey–Kramer multiple comparison test, *p* < 0.05). (**d**) Pie chart and bar representation of Cd mass percentage in each fraction of the midgut gland extract of the oil layer, supernatant, and precipitate. Error bars in the bar graph represent the standard deviation (n = 3).

In the search for Cd-binding substances in scallops, the scallop tissues were separated into five parts, including the midgut gland, gonad, mantle, gills, and adductor muscle, to confirm which tissue contained a large amount of Cd (Fig. 1a). Part of the tissue was decomposed with nitric acid, and the Cd concentration was measured using ICP-OES. As a result, the Cd concentration in scallops was significantly high in the midgut gland, followed by the mantle, gills, adductor muscle, and gonads (Fig. 1b). The Cd concentration in the midgut gland per tissue weight was also significantly high, followed by the mantle, gonad, gills, and adductor muscle (Fig. 1c). The most accumulated Cd was observed in the midgut gland, which accumulated 672.10 µg of Cd per the whole tissue, and the Cd concentration in the midgut gland was 143.00 µg/g. These results suggested that the midgut gland of scallops contained Cd-binding substances. Therefore, we investigated Cd-binding substances in the midgut glands of scallops.

### Measurement of the metal concentration in the three layers of the midgut gland extract

To identify Cd-binding substances in the midgut gland, organic molecules were extracted using a buffer. The scallop midgut gland extract was divided into three layers: the oil layer, the supernatant, and the precipitate. Then, the metal concentration in each fraction was measured using ICP-OES. The oil layer contained 1%, the supernatant contained 84%, and the precipitate contained 15% of total Cd (Fig. 1d). The Cd-rich supernatant fraction likely contained water-soluble organic molecules, such as proteins and sugars.

### Detection of Cd-binding protein

#### HPLC combined with post-column TPPS method

Cd-binding substances in scallops were abundant in the water-soluble fraction of the midgut gland. If the water-soluble fraction contained a high concentration of Cd, Cd was likely coordinated by a hydrated complex, highly polar organic polymer such as a protein, or small hydrophilic organic molecule. The metal is generally bound to organic molecules in living tissues or body fluids. Therefore, we first confirmed whether Cd was an organic polymer, small hydrophilic organic molecule, or hydration complex using the post-column method. When the water-soluble fraction was subjected to gel filtration chromatography, the Cd-containing fraction was separated by molecular weight. After separation by gel filtration chromatography, TPPS was mixed with each separated fraction to detect the Cd-containing fraction. The absorption wavelength of TPPS changed from 414 to 432 nm upon coordination with Cd, and the presence of Cd-bound molecules was detected by the absorption wavelength change in TPPS.

**Fig. 2:**
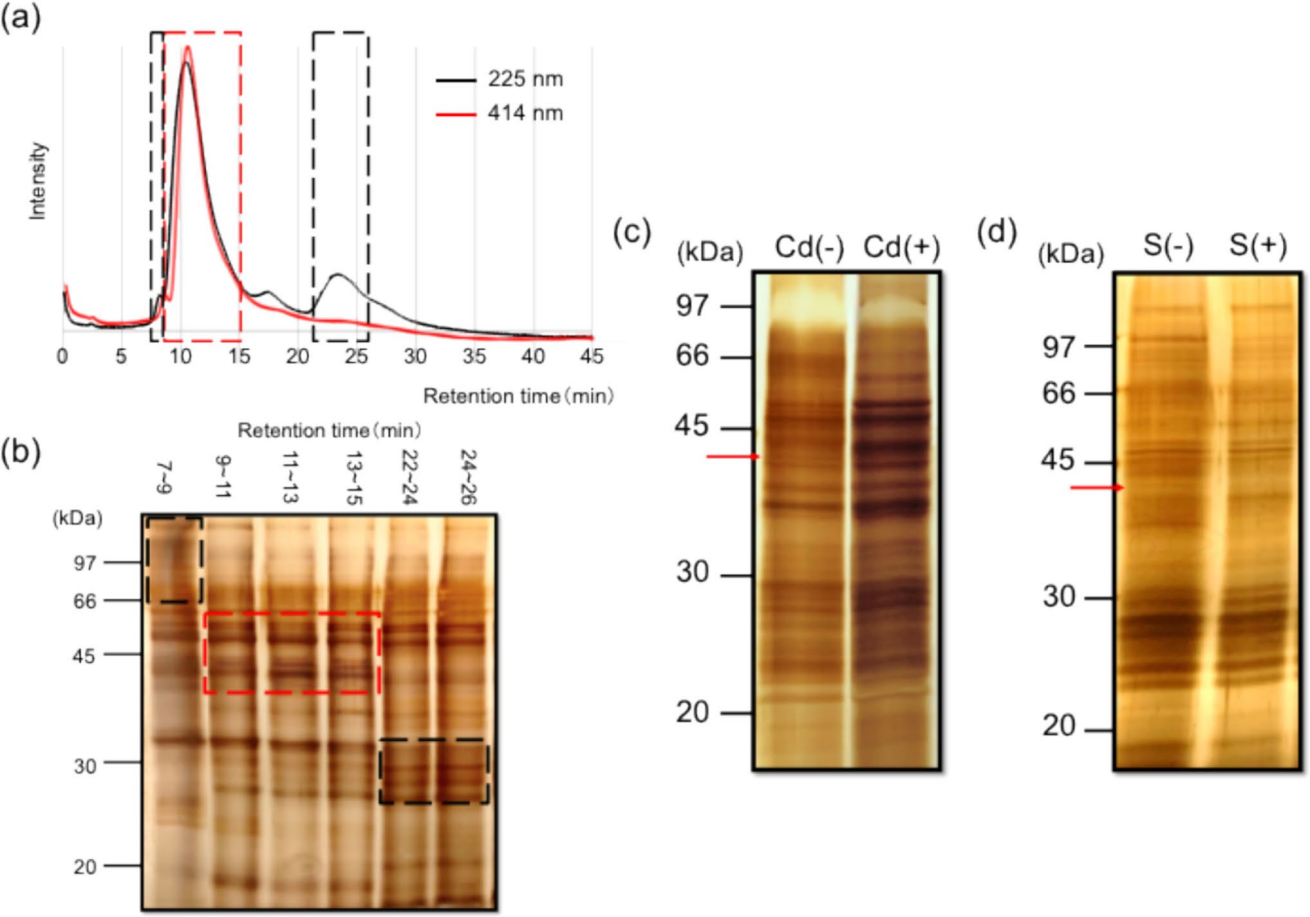
Detection and identification of Cd-binding proteins via the HPLC combined with post-column TPPS, the Cd binding competitive inhibition, and the CdS precipitation experiments. (**a**) The supernatant of the midgut gland extract was separated via HPLC using a gel filtration column with post-column TPPS detection. The black line is the signal detected at 225 nm, indicating the presence of proteins. The red line is the signal detected at 414 nm, indicating the presence of free TPPS. (**b**) SDS-PAGE analysis of proteins fractionated by HPLC. The supernatant of the midgut gland extract was separated by the gel filtration column without flowing the post-column solution, fractionated at 1-min intervals. Three distinct sets of fractions were collected: fractions from 7–9, 9–15, and 22–26 min. (**c**) SDS-PAGE analysis on the Cd-immobilized affinity chromatography experiment on the midgut gland extract. Cd (−) and Cd (+) represent without and with Cd, respectively. (**d**) SDS-PAGE analysis on the CdS precipitation experiment on the midgut gland extract. S (−) and S (+) represent only Cd, and both Cd and S added, respectively. The red arrow indicates the target protein band with high reproducibility.

The UV/vis absorption at 414 nm, which is the absorption maximum of free TPPS, was observed at retention times of 9–15 min, suggesting the presence of Cd-binding substances (Fig. 2a). The fractions from 9 to 15 min were fractionated every 1 min. Each fraction was subjected to SDS-PAGE to detect specific proteins. For comparison, fractions from 7–9 min and 22–26 min that did not exhibit absorption at 414 nm were collected and subjected to SDS-PAGE as negative controls. Results revealed the specific protein bands were detected at approximately 35–66 kDa. However, several protein bands overlapped on the gel. The sequences of proteins that bound to Cd were difficult to identify (Fig. 2b).

#### Cd-immobilized affinity chromatography method

Organic polymer compounds with a molecular weight of 35–66 kDa were likely Cd-binding substances as a result of the HPLC combined with post-column TPPS experiment. However, as several protein bands were observed at 35–66 kDa, we could not identify the Cd-binding proteins. Therefore, as another method, the Cd-immobilized affinity chromatography experiment was conducted, in which Cd was immobilized on a resin. Excessive addition of Cd to Cd-binding substances in scallops formed Cd-complex in solution. After the formation of the Cd-complex, it did not bind to the Cd-immobilized in the affinity column, and all the Cd-complexes in the solution passed through the affinity column. Utilizing these properties, the protein band pattern of the eluate should change depending on the presence or absence of excess Cd added to the midgut gland extract. We attempted to identify the target substance by comparing changes in SDS-PAGE images (Fig. 2c). Several bands disappeared with the addition of Cd. After repeating this experiment, a band at approximately 44 kDa disappeared with the addition of excess Cd with high reproducibility (arrow in Fig. 2c). The identification of the 44 kDa protein was consistent with the HPLC combined with post-column TPPS experiment, which refined the molecular weight of Cd-binding proteins to the range of 35–66 kDa.

#### CdS precipitation method

To ensure the reproducibility of Cd-binding protein identification, an additional experiment was conducted using the CdS precipitation method. The Cd-binding substance bound to Cd to form a Cd-complex. The addition of S^2−^ induced the co-precipitation of both CdS and the Cd-complex because the solubility product of CdS was low in the solution. Cd-binding proteins were investigated by comparing the changes in protein contents in the supernatant of the midgut gland extract, with and without the addition of S^2−^ by SDS-PAGE (Fig. 2d). The protein band pattern of SDS-PAGE showed that a protein band around 44 kDa disappeared upon the addition of S^2−^, indicating that 44 kDa proteins bound to Cd in solution (arrow in Fig. 2d).

### LC–MS/MS analysis

Proteins with a molecular weight of 44 kDa were detected as candidates for Cd-binding proteins. Therefore, we performed LC–MS/MS analysis to determine the amino acid sequence. Tables 1 and 2 show the LC–MS/MS results from the Cd-immobilized affinity chromatography, and the CdS precipitation experiment, respectively.

**Table 1:** Proteins estimated by LC–MS/MS from the Cd-immobilized affinity chromatography experiment. The 10 proteins with the highest Sum PEP scores are listed.

Accession	Protein	Sum PEP Score	Coverage	MW [kDa]
XP_021356192.1	meprin A subunit alpha-like	33.41	46.68	44.3
XP_021372089.1	tubulin beta-4B chain-like isoform X1	28.49	41.57	49.8
XP_021345391.1	tubulin beta-4B chain-like	26.34	37.08	49.8
XP_021372096.1	tubulin beta chain-like	22.53	30.70	49.5
XP_021371808.1	1-aminocyclopropane-1-carboxylate synthase-like protein 1	20.53	35.46	51
XP_021376587.1	actin, cytoplasmic 1-like	17.62	21.07	41.6
XP_021352421.1	tubulin alpha-1A chain-like	17.57	26.39	50.1
XP_021356759.1	actin-2-like	15.80	31.12	41.7
XP_021369414.1	uncharacterized protein LOC110460687	12.11	18.40	36.7
XP_021376916.1	4-hydroxyphenylpyruvate dioxygenase-like	11.27	21.99	43.8

**Table 2:** Proteins estimated by LC–MS/MS from the CdS precipitation experiment. The 10 proteins with the highest Sum PEP scores are listed.

Accession	Protein	Sum PEP Score	Coverage	MW [kDa]
XP_021350524.1	xylose isomerase-like	50.62	41.30	51.1
XP_021356192.1	meprin A subunit alpha-like	44.63	48.72	44.3
XP_021361175.1	putative aminopeptidase W07G4.4 isoform X1	32.72	31.60	56.1
XP_021379046.1	xanthine dehydrogenase-like	30.71	15.43	146.2
XP_021348303.1	sushi domain-containing protein 2-like isoform X1	29.88	17.51	120.1
XP_021342314.1	cystathionine gamma-lyase-like	22.59	27.59	43.5
XP_021356263.1	leucine-rich repeat-containing protein let-4-like	22.20	24.90	53.3
XP_021355139.1	collagen alpha-4(VI) chain-like	21.04	6.24	148.5
XP_021354704.1	glutamate dehydrogenase, mitochondrial-like	13.50	19.37	59.5
XP_021377931.1	uncharacterized protein LOC110466016 isoform X1	13.25	3.71	466.4

We then focused on XP_021356192.1 in terms of the molecular weight and reproducibility. LC–MS/MS identified *M. yessoensis* meprin A subunit alpha-like (myMEP1A), a protein with a total length of 44.3 kDa and an N-terminal signal peptide. myMEP1A belongs to the peptidase family M12A and has a C-terminal transmembrane domain. The sequence of myMEP1A is shown in Fig. S1.

### Tissue-specific expression analysis of myMEP1A

To confirm the expression levels of myMEP1A, total RNA was extracted from each scallop tissue (the adductor muscle, gill, mantle, midgut gland, and gonad) and the first-strand cDNA was synthesized. PCR was performed to confirm the mRNA expression level of myMEP1A at each tissue.

**Fig. 3:**
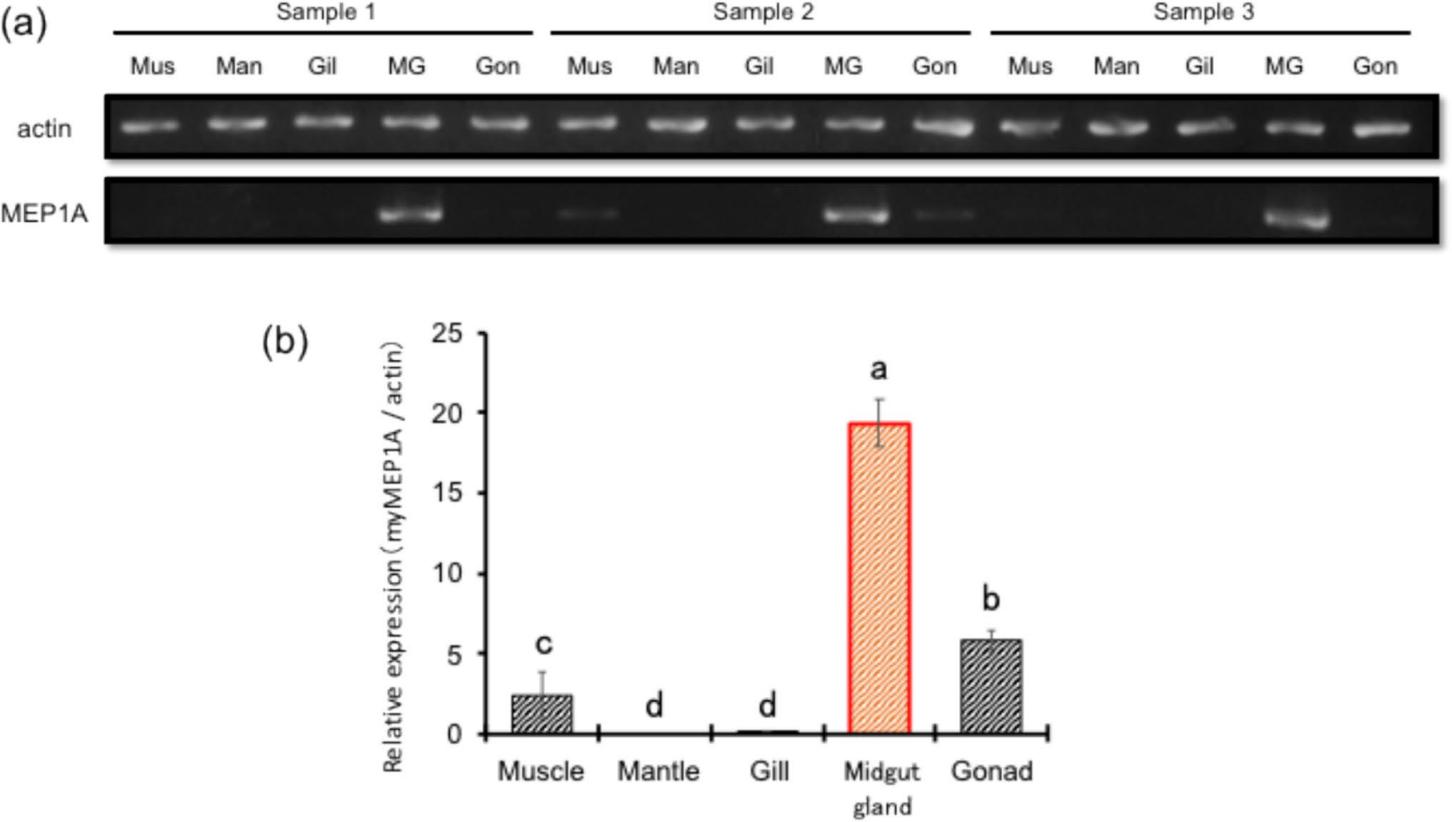
Tissue-specific expression of myMEP1A using PCR analysis. (**a**) Agarose gel electrophoresis of the PCR products, amplified from the adductor muscle, mantle, gill, midgut gland, and gonad cDNA. Actin was used as a housekeeping gene. (**b**) Relative expression levels analyzed from the band intensity using ImageJ software. Error bars represent the standard deviation (n = 3). Different alphabets above the bar (e.g., a–d) represent statistical differences (Tukey–Kramer multiple comparison test, *p* < 0.05).

The agarose gel electrophoresis results are shown in Fig. 3a. The mRNA expression levels of myMEP1A and actin, a housekeeping gene, were determined. Relative quantification using ImageJ software revealed that myMEP1A was significantly expressed at high levels in the midgut gland (Fig. 3b).

### Preparation and purification of recombinant myMEP1A (r-myMEP1A)

pET-44a (+) vector with a myMEP1A sequence was transformed to *E. coli* BL21 competent cells. For efficient recombinant protein production, the IPTG induction condition was optimized. IPTG induction was performed at 37 °C for 2 h, 16 °C for 7 h, and 5 °C for 24 h with an IPTG concentration of 10, 100, and 1000 µM. The cells were harvested and sonicated. The soluble and insoluble fractions were subjected to SDS-PAGE analysis (Fig. S2). The target protein was stained near 99 kDa. From the band intensity, we determined the optimal condition with 1 mM IPTG at 37 °C for 2 h.

**Fig. 4:**
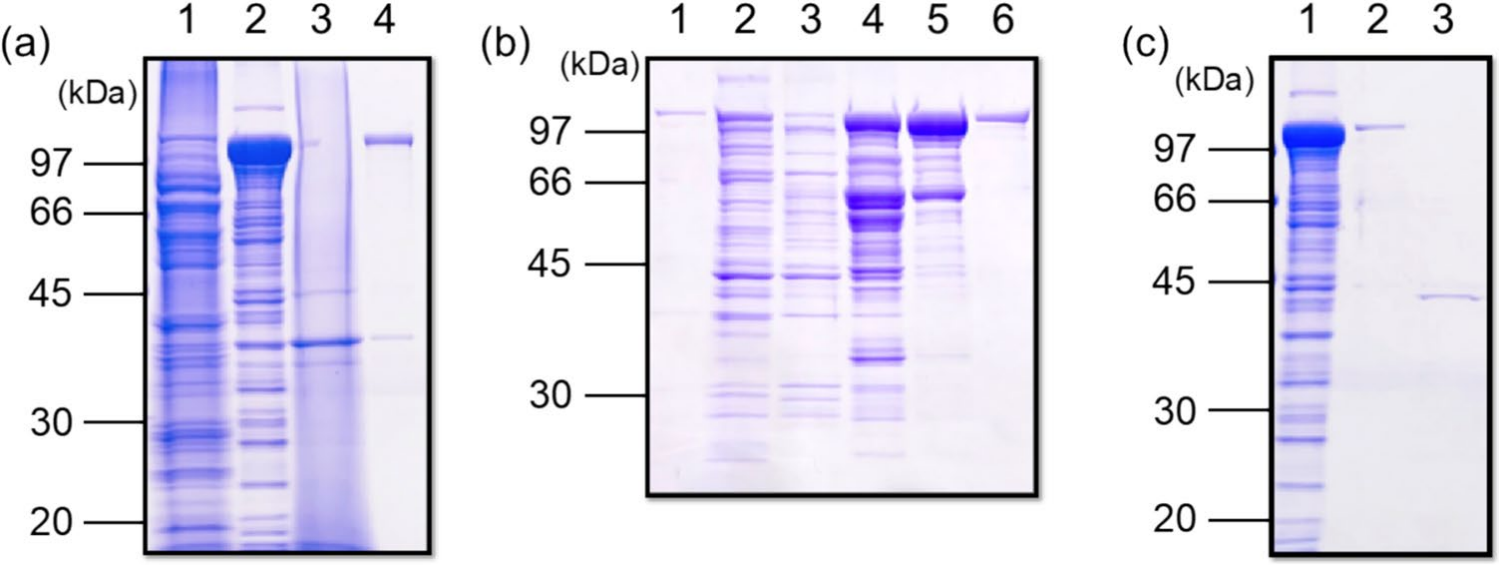
Proteins during expression and purification of r-myMEP1A. (**a**) SDS-PAGE of soluble fraction after sonication of *E. coli* cells with pET-44a (+) empty vector (lane 1) and pET-44a (+)-myMEP1A vector (lane 2), and insoluble fraction with pET-44a (+) empty (lane 3) and pET-44a (+)-myMEP1A vector (lane 4). (**b**) Ni-affinity chromatography for purification of r-myMEP1A with flow through (lane 1), eluate with 20 mM imidazole (lane 2), 50 mM imidazole (lane 3), 100 mM imidazole (lane 4), 300 mM imidazole (lane 5), and 500 mM imidazole (lane 6). (**c**) Tag-removal of r-myMEP1A with soluble fraction after sonication of *E. coli* cells with pET-44a (+)-myMEP1A vector (lane 1), r-myMEP1A before thrombin digestion (lane 2), and r-myMEP1A after thrombin digestion (lane 3).

The soluble and insoluble fractions were analyzed using SDS-PAGE and compared to those using pET-44a (+) empty vector (Fig. 4a). Ni-affinity chromatography was performed to purify r-myMEP1A (Fig. 4b), which was successfully purified in the solution eluted with 500 mM imidazole, followed by His-tag cleavage using thrombin (Fig. 4c).

### Binding experiment of Cd and r-myMEP1A

**Fig. 5:**
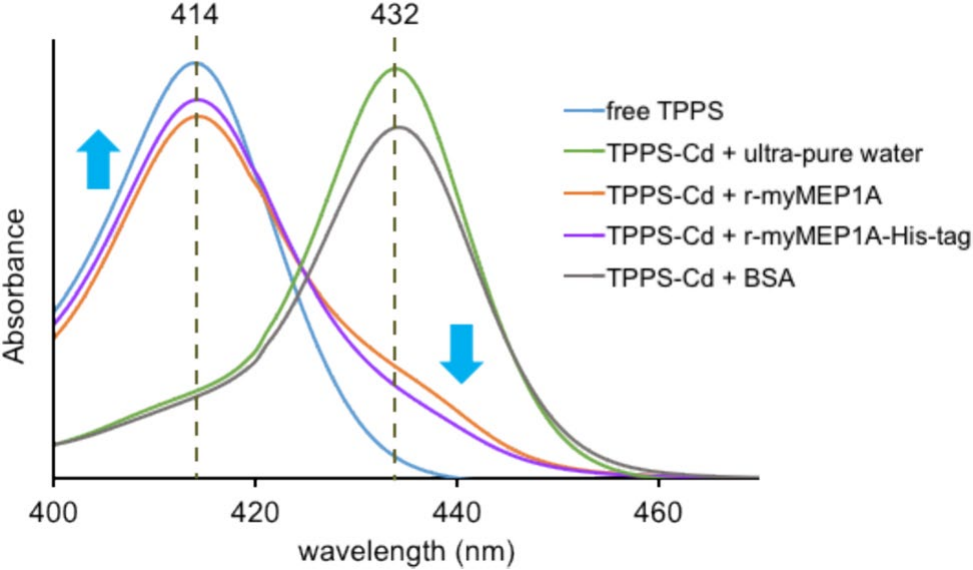
Binding experiment of Cd and r-myMEP1A using TPPS as an indicator. The spectral lines are as follows: blue line: free TPPS; green line: TPPS-Cd complex; orange line: r-myMEP1A and TPPS-Cd complex; purple line: r-myMEP1A with His-tag and TPPS-Cd complex: grey line: BSA and TPPS-Cd complex.

The binding affinity of r-myMEP1A to Cd was investigated using TPPS. The maximum absorption wavelengths were 414 nm for free TPPS and 432 nm for TPPS-Cd complex. When Cd-binding substances were mixed with TPPS, TPPS-Cd complex was not formed. Then, Cd-binding substances caused the maximum absorption at 414 nm. Figure 5 represents the UV/Vis spectrum of the TPPS solution. We observed the binding of r-myMEP1A to Cd, as evidenced by increased and reduced absorbances at 414 nm and 432 nm, respectively, indicating the presence of free TPPS. Additionally, from the changes in the maximum absorption wavelengths of 414 and 432 nm, we observed that r-myMEP1A with His-tag exhibited stronger binding affinity. Moreover, BSA, used as a negative control, demonstrated weak binding affinity. These results confirmed that r-myMEP1A bound to Cd by displacing it from the TPPS-Cd complex, subsequently forming its own bond with Cd.

### Cd-binding site prediction and sequential alignment of myMEP1A

The tertiary structure of myMEP1A was modelled using the SWISS-MODEL server (Fig. S3).As a template for modelling myMEP1A, 7UAE contained two calcium ions, coordinated mainly by the oxygen atoms in aspartic acid residues and the main chain in the MAM domain, and a zinc ion coordinated by three histidine residues in the astacin domain. The modelled structure of myMEP1A lacked the three histidine residues core in the astacin domain by containing Q122, Y126, and N132 at the Zn-missing site. Conversely, the two metal-binding motifs in the MAM domain were conserved. We denoted these two metal-binding motifs as Cd-binding sites 1 and 2. Cd-binding site 1 contained the oxygen atoms of the main chain in D252 and F257 and the side chains in D255 and D258. Cd-binding site 2 contained the oxygen atoms of the main chain in T241 and Y283 and the side chains in E243, T271, and D381.

The amino acid sequence of myMEP1A was aligned with MEP1A of other molluscs using CLUSTALW (Fig. S4). Cd-binding sites 1 and 2 were highly conserved in *Argopecten irradians*, *Magallana gigas*, *Ostrea edulis*, *Pecten maximus*, and *Ylistrum balloti*. Aspartate was substituted with asparagine only in Cd-binding site 1 of *Mytilus edulis*. Moreover, the lack of three histidine at the Zn-missing site was conserved in all the molluscan species investigated in this study.

## Discussion

To elucidate Cd accumulation in scallops, we quantified Cd concentrations in various scallop tissues, including the midgut gland, gonad, gill, mantle, and adductor muscle. Our findings revealed significant variations in Cd concentrations across the different tissues. Notably, the adductor muscle exhibited comparatively lower Cd concentrations, whereas the midgut gland exhibited higher Cd concentrations, as reported previously (Luo et al. 2014). This result coincided with the previous report that the midgut gland comprises approximately 10% of the total body weight and can accumulate up to approximately 65% of the total Cd content (Ikuta 1985). Cd is taken up from seawater, transported to the midgut gland, and accumulated in the presence of Cd-binding substances. These Cd-binding substances facilitate Cd accumulation at low concentrations (typically ~ 0.02 µg/L in unpolluted waters) to reach concentrations approximately one million times higher (Mart and Nürnberg 1986). Given the extensive Cd accumulation observed in the midgut gland, scallops may possess mechanisms that either detoxify or effectively utilize Cd in their physiological processes.

In the present study, the chemical forms of Cd in the midgut gland were determined. The ICP-OES measurements of metal concentrations in each layer of the scallop tissue extract revealed that the minimum Cd concentration was in the lipid-soluble components, whereas the water-soluble fraction exhibited a comparatively higher Cd concentration. This result was consistent with that of the previous research on Cd in various foodstuffs, such as rice, crabs, and squid (Jennings et al. 1979; Santoso et al. 2013). Previous studies on rice have shown high Cd concentrations in water-soluble polar components and proteins (Zhang et al. 2009). The substantial Cd in the water-soluble fraction indicated the presence of Cd complexes solubilized by various molecules.

To separate the midgut gland extract according to the molecular weight, we performed HPLC on the gel filtration column combined with the post-column TPPS detection. This approach facilitated the investigation of the Cd-complex in the water-soluble fraction, including a hydrated complex, highly polar organic polymer, and small hydrophilic organic molecule. Our findings indicated that Cd-binding substances predominantly comprised macromolecular compounds ranging in size from approximately 35 to 66 kDa. This finding strongly suggested that high-molecular weight proteins coordinated to Cd^2+^. Smaller molecules with lower binding constants may have been replaced with hydration complexes during the extraction process and Cd was subsequently released into the water-soluble fraction. Although extensive research has focused on metal-binding compounds, high-molecular weight Cd-binding substances in scallops have not been previously identified, unlike well-known low-molecular weight metal-binding substances, such as siderophores and metallothionein (Amiard et al. 2006; Leong 1986). Siderophore and metallothionein are widely known as metal chelators in various organisms, demonstrating their importance in metal homeostasis and detoxification mechanisms (Cherian and Nordberg 1983; Wang 2002). The lack of previous literature on the use of high-molecular weight Cd-binding substances in organisms, particularly in scallops, emphasizes the novelty of this finding.

In the Cd-immobilized affinity chromatography experiment, we identified 44 kDa proteins that consistently displayed high reproducibility across multiple experiments. To ensure the reproducibility of the protein identification, an additional experiment was conducted using CdS precipitation. We consistently observed the disappearance of the 44 kDa band when Cd and S were combined, providing strong evidence for the involvement of this protein in Cd-binding processes. Remarkably, three Cd-binding protein detection methods consistently revealed that 44 kDa protein in the midgut gland extract caused Cd-binding.

After performing LC–MS/MS analysis and organizing the results by score, the highest-scoring protein from the Cd-binding experiments was identified as myMEP1A. Despite the highest score in xylose isomerase-like protein from the CdS precipitation experiment, its molecular weight of 51.1 kDa was not consistent with the SDS-PAGE results. The second highest-scoring protein from the CdS precipitation experiment was also myMEP1A, which had an expected molecular weight of 44.3 kDa, aligned with the band position, and demonstrated high reproducibility. Therefore, we concluded that myMEP1A was the likely candidate of the Cd-binding substance. Regarding xylose isomerase-like compounds, contamination from adjacent bands or other sample interference during the measurements may have influenced the results. myMEP1A is a full-length protein, with a molecular weight of 44.3 kDa. Notably, previous research has reported the existence of a Cd-binding substance within a high molecular weight protein of approximately 45 kDa, which aligned with our findings (Fowler and Megginson 1986). MEP1A plays a role in cleaving several basement membrane components, contributing to epithelial differentiation and cell migration (Schütte et al. 2007). Meprin is a multi-subunit metallopeptidase found in the kidneys of rodents and human intestines and is responsible for cell proliferation, cell migration, developmental pattern formation, and tissue construction (Sterchi et al. 2008). The alpha and beta subunits of meprin can form either soluble alpha2 homodimers or membrane-bound alpha/beta heterodimers via disulfide bonds (Chevallier et al. 1996). myMEP1A possesses two domains including the astacin superfamily domain and the MAM domain. The astacin-like subfamily, or peptidase family M12A, is a group of protein-degrading enzymes with HExxH metal-binding/active sites (Bond and Beynon 1995). However, myMEP1A does not possess the conserved metal-binding active site typically found in the astacin family. The high similarity in the overall sequence of myMEP1A resulted in its classification as a metalloendopeptidase. The MAM domain, found in a diverse range of protein families, is an extracellular domain that mediates protein–protein interactions responsible for cell adhesion (Fujimura et al. 2006). The meprin alpha subunit tends to self-aggregate. Once secreted, the meprin alpha subunit forms large multimers with molecular weights of > 1,000 kDa.

In this study, we elementally analyzed various scallop tissues, revealing a notably high Cd concentration in the midgut gland. To elucidate the functional roles of myMEP1A in scallops, we investigated its mRNA expression levels in different tissues. Our findings demonstrated the significant expression of myMEP1A in the midgut gland, which coincided with the Cd accumulation site. This correlation suggested the potential involvement of myMEP1A in Cd accumulation in scallops. A previous study in humans highlighted MEP1A as a secretory metalloproteinase capable of cleaving numerous protein substrates, some of which are closely associated with cancer (Broder and Becker-Pauly 2013). Extensive MEP1A expression has been linked to hepatocellular carcinoma, promoting the migration and infiltration of cancer cells (OuYang et al. 2016).

The ability of r-myMEP1A to bind Cd was crucial in our study. We used the wavelength changes observed from free TPPS and TPPS-Cd complex as indicators. The solution containing r-myMEP1A, Cd and TPPS revealed an increase in the absorbance at 414 nm, corresponding to the absorption maximum of free TPPS, and a decrease in absorbance at 432 nm, corresponding to the absorption maximum of TPPS-Cd. These results strongly indicated that r-myMEP1A effectively competes with TPPS-Cd for Cd binding. Thus, myMEP1A exhibited a strong capacity for Cd-binding.

The homology-modelling of myMEP1A demonstrated the metal-binding site conserved in the MAM domain and that lacking in the astacin domain. Amino acids which possess oxygen atoms coordinated to metal ions in the side chain were evolutionarily conserved among molluscan species. Two possible Cd-binding sites in the MAM domain contributed to the Cd-binding capacity of myMEP1A. Owing to the absence of the zinc-binding motif in the astacin domain, myMEP1A may not possess peptidase activity.

Collectively, our findings provided a comprehensive view of Cd accumulation in *M. yessoensis*. The co-localization of high Cd concentration and high expression of myMEP1A in the midgut gland, along with LC–MS/MS identification of myMEP1A as a Cd-binding protein, supported the functional role of myMEP1A in Cd detoxification. The Cd-binding ability of r-myMEP1A revealed by TPPS measurements, and the conserved metal-binding residues in the MAM domain, suggested its involvement in Cd accumulation. Seawater naturally contains trace levels of Cd, which scallops acquire through filter feeding. The absorbed Cd was transported to the midgut gland, where myMEP1A bound to Cd^2+^. This Cd-binding facilitated Cd accumulation and facilitated its detoxification (Fig. S5).

The identification of the high-molecular weight Cd-binding protein in scallops may lead to new research on metal-binding and detoxification in marine organisms. Understanding the Cd-binding mechanism of this protein may contribute to the development of novel biomaterials for Cd adsorption in polluted areas, as well as new biomarkers for monitoring Cd concentrations in marine environments.

## Conclusion

We identified myMEP1A as a Cd-binding protein in the midgut gland of scallops. Our findings suggested that myMEP1A plays a crucial role in the binding and storage of Cd in the midgut glands of scallops. This study presented an initial step towards understanding the Cd-binding mechanism in the midgut glands of *M. yessoensis*. Future research should aim to determine the extent to which myMEP1A contributes to Cd binding in *M. yessoensis*, elucidate its binding mechanism from a structural perspective, and analyze the myMEP1A expression profile upon Cd exposure. Additionally, we explored the presence of more potent Cd-binding proteins within these organisms. These investigations will not only expand our understanding of Cd sequestration mechanisms in scallops but may also provide valuable insights into broader metal homeostasis processes in marine organisms.

## Supplementary Information

Below is the link to the electronic supplementary material.

Supplementary file1 (PDF 842 KB)

## Data Availability

Data will be provided upon request of the corresponding author.
