# Supplementary material for: Exploring cadmium-binding proteins in Japanese scallops Mizuhopecten yessoensis: isolation and characterization
Source: Environ Sci Pollut Res Int. 2025 Aug 5;32(31):18961–74. doi: 10.1007/s11356-025-36799-1 (PMC12357810; doi:10.1007/s11356-025-36799-1)
Supplement: Supplementary file 1 — Supplementary file1 (PDF 842 KB) [file 11356_2025_36799_MOESM1_ESM.pdf]

Supplementary Information for

Exploring cadmium-binding proteins in Japanese scallops *Mizuhopecten yessoensis*: isolation and characterization

Zehua Zheng[1,2†], Yuto Namikawa[1†], Yugo Kato[1], Peng Lu[1,3], Lumi Negishi[4], Hitoshi Kurumizaka[4], Koji Nagata[1], Michio Suzuki[1*]

[1]Department of Applied Biological Chemistry, Graduate School of Agricultural and Life Sciences, The University of Tokyo, 1-1-1 Yayoi, Bunkyo-ku, Tokyo, 113-8657, Japan.

[2]Bone Marrow Transplantation Center of the First Affiliated Hospital & Liangzhu Laboratory, Zhejiang University School of Medicine, Hangzhou, China.

[3]Future Food Laboratory, Innovation Center of Yangtze River Delta, Zhejiang University, No. 828, Zhongxing Road, Xitang Town, Jiashan County, Jiaxing City, Zhejiang Province, China.

[4]Institute for Quantitative Biosciences, The University of Tokyo, 1-1-1 Yayoi, Bunkyo-ku, Tokyo, 113-0032, Japan.

[†]These authors contributed equally to this work

*Corresponding author: Michio Suzuki

E-mail: amichio@g.ecc.u-tokyo.ac.jp

Table S1 Sequences of primers used for quantification of mRNA expression levels.

Primer sequence	
Actin	
F:	TATGAGATGAAGCCCAGA
R:	AGAAGGAATGGCTGGAATAGGGAT
myMEP1A	
F:	AGCAATGAGGATAACTAC
R:	GTTCCCAGTCACAGAAGTTA

Table S2 Sequences of primers used in In-Fusion reaction.

Primer sequence (for pET44a (+) vector linearization)	
F:	GGTTCTGGTAATTGATGA
R:	CGCGCTGCCCCGGGGGACCA
Primer sequence (for myMEP1A gene amplification)	
F:	CCCGGGCAGGTTCCAGTCAGAAG
R:	AATTACCAGAACCTTACACCAGGCATTTACCAC

1 MDARLILILA LTSCCLAVPQ RQKRNILTDK NRLWPFGVVP VVFDKSIDGF TLEKILASMQ

61 EVQMSTFSAS RSCLAFVPRS NEDNYALFTV VPGVSGDGEP GMSGGAQTIH LYPNMAKADI

121 MQLLVYTLGF YNEFRRPDRE NYVTVNMDNI AEKDQKYFKI GNTTTFYNYP FDFKSITFFY

181 PYAYAKDPSK PTIQARYESQ VFPWKVSLSS FDVSNLQRIY ECGTDTSNRL DLLSGMISKC

241 TFEFNFCDWE QDTADDFDLE RHMGQSSSEE TGPQADFSSG IGYYALAGAK NHHNSATRLI

301 SPELPAGDYC MRFHYYMYGS DVRKARLVRR MGGNDEVLAE IEGNQGNLWH RYSETISSPD

361 NFKFVLEAMT GGSDLGDIAI DDVYILRGKC LV

Fig. S1 Amino acid sequence of myMEP1A (yellow: astacin superfamily domain, green: MAM domain, black line: peptide detected by LC-MS/MS analysis, dotted line: signal peptide)

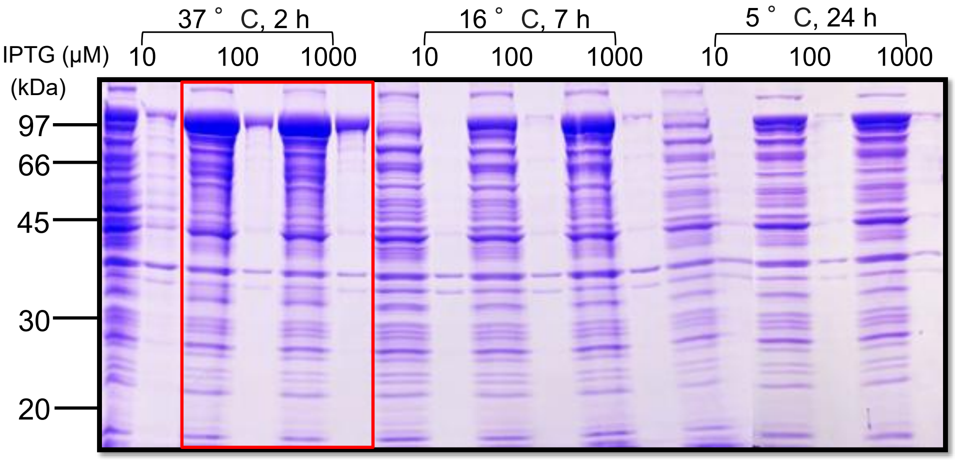

Fig. S2 Optimization of large-scale expression conditions for r-myMEP1A. Each pair of adjacent lanes represents soluble (left) and insoluble (right) fractions, respectively. The optimal condition was as follows: 1 mM IPTG at 37°C for 2 h.

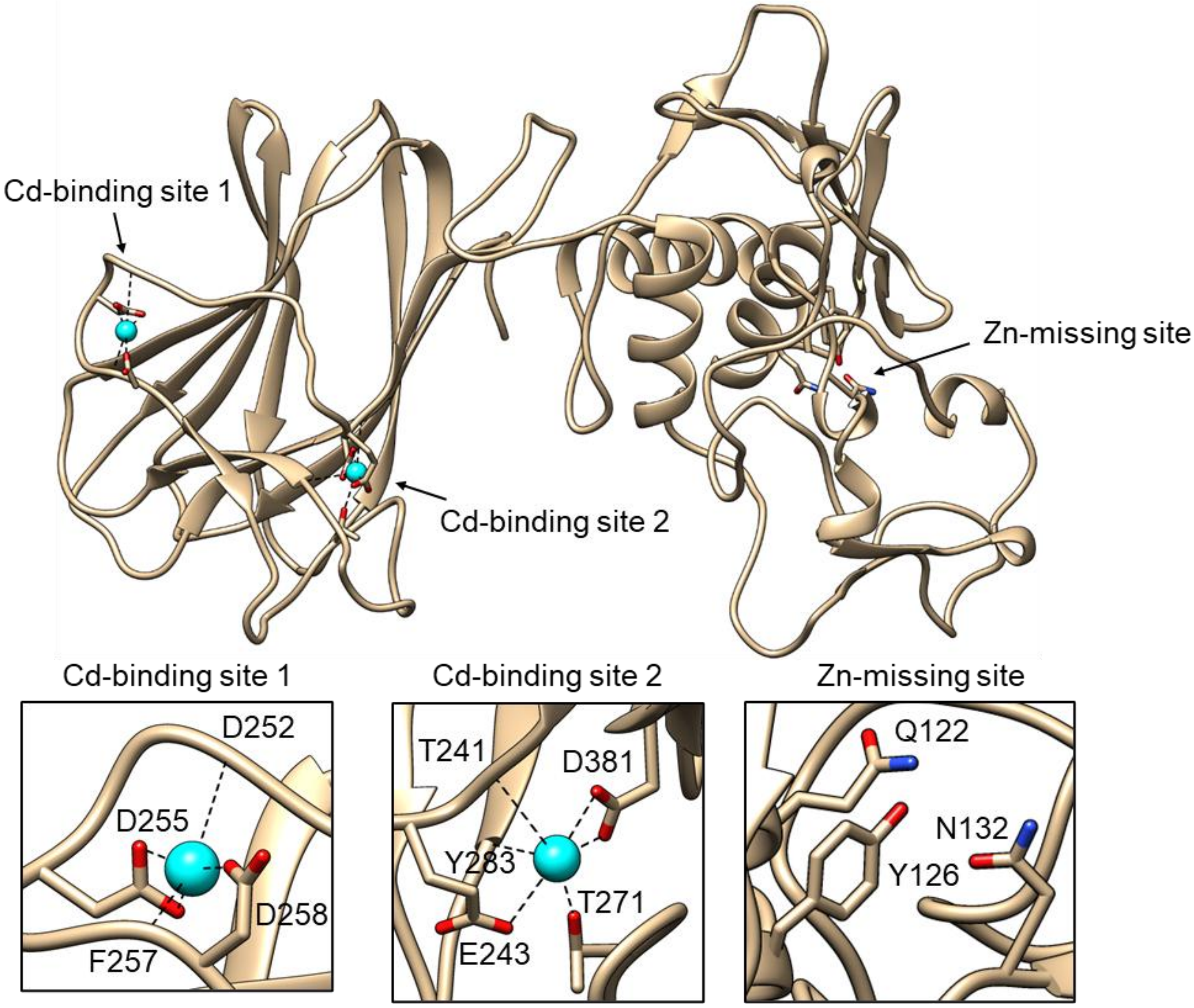

Fig. S3 Modelled structure of myMEP1A using SWISS-MODEL. The left and right domains are the MAM domain and the astacin domain, respectively. The cadmium ions were placed in the same place as the calcium ions in 7UAE. Cyan spheres represent cadmium ions. In the Cd-binding site 1, D252 and F257 in the main chain and D255 and D258 in the side chain coordinate to the cadmium ion. In the Cd-binding site 2, T241 and Y283 in the main chain and E243, T271 and D381 in the side chain coordinate to the cadmium ion. In the Zn-missing site, Q112, Y126 and N132 are present instead of histidine. Dotted lines represent the hypothetical coordination bond between the oxygen atoms and the cadmium ions.

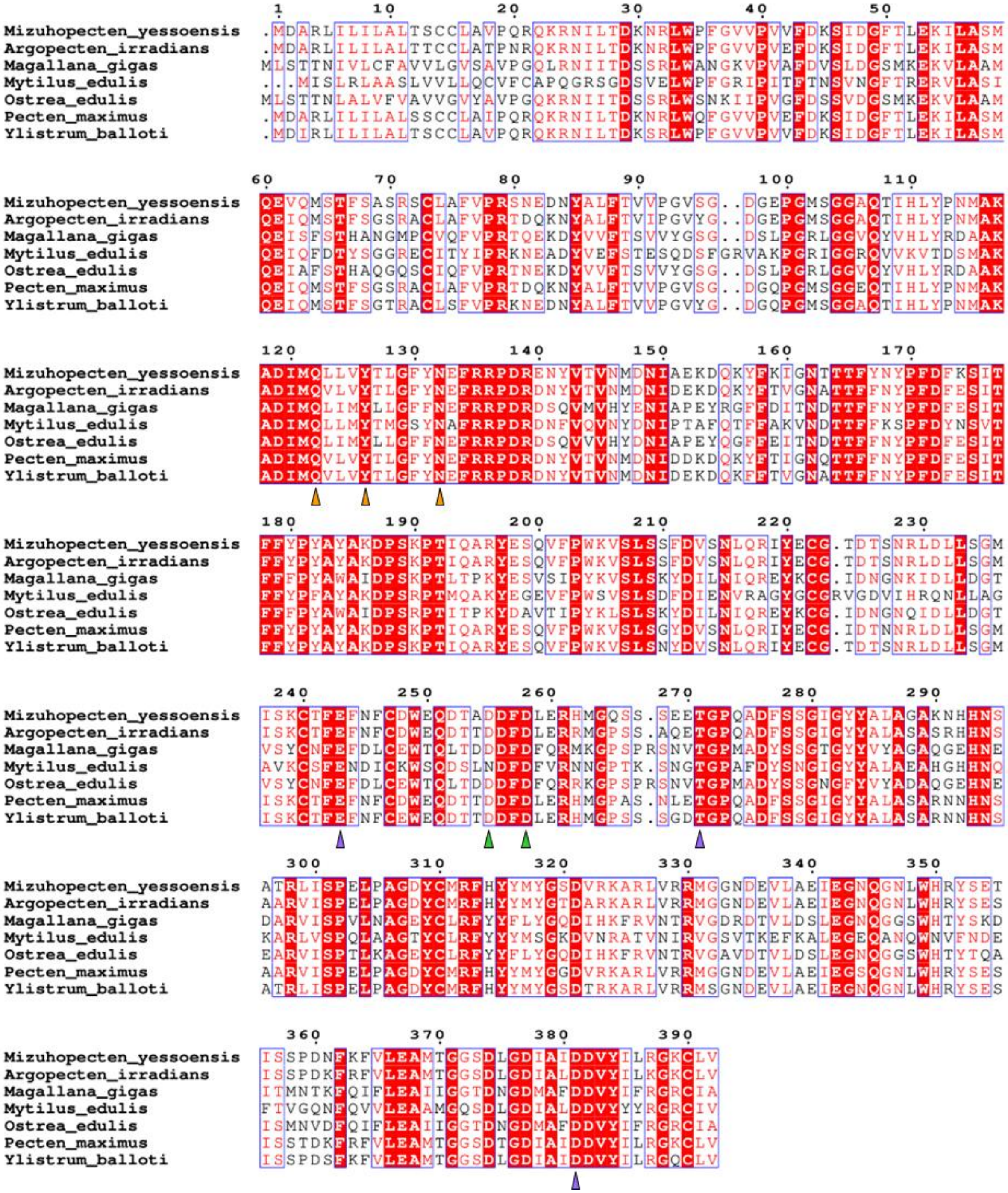

Fig. S4 Alignment of myMEP1A with MEP1A (meprin A subunit alpha-like) of other molluscs. Multiple sequence alignment was performed using CLUSTALW. The aligned amino acid sequences were visualized using ESPript 3.0. The green, purple and orange arrows indicate Cd-binding site 1, Cd-binding site 2, and Zn-missing site.

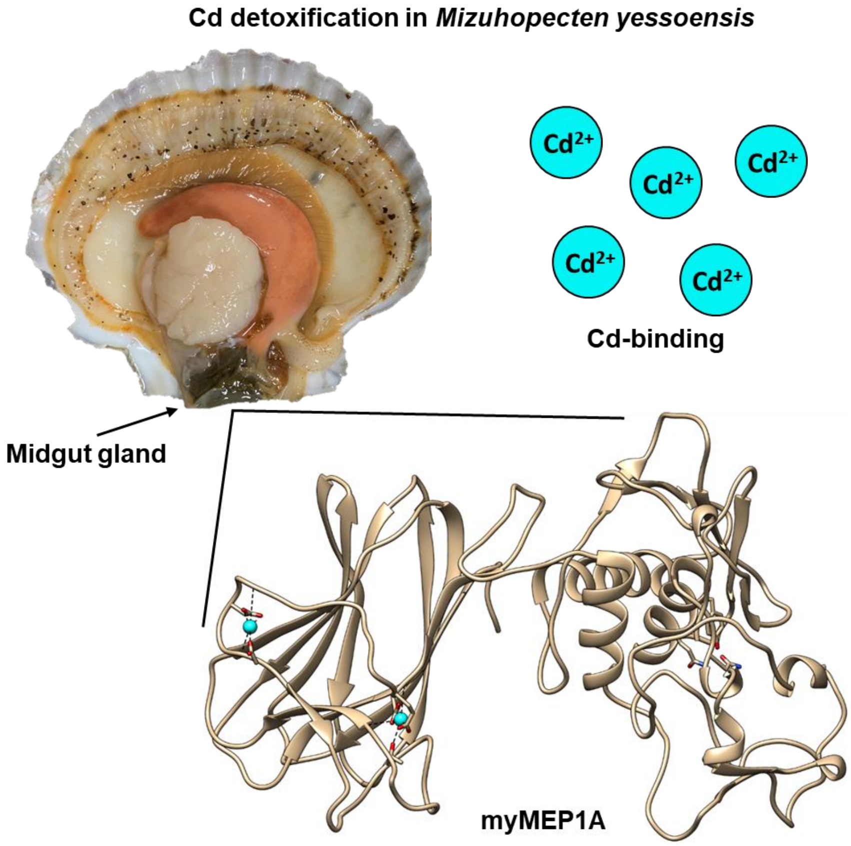

Fig. S5 Schematic representation of Cd detoxification by myMEP1A in *M. yessoensis*. The midgut gland accumulates Cd that is taken up through filtering seawater. myMEP1A, a metal-binding protein specific to the midgut gland, binds to Cd^{2+} to mitigate heavy metal toxicity.
